# Evaluation of the Prevalence of Genetic Variants at the Nebulette Locus in Cavalier King Charles Spaniels

**DOI:** 10.3390/ani16020298

**Published:** 2026-01-19

**Authors:** Caroline Melis, Claire Wade, Claudia Rozendom, Frank G. van Steenbeek, Niek J. Beijerink

**Affiliations:** 1 Veterinaire Specialisten Vught, Reutseplein 3, 5264 PN Vught, The Netherlands; c.melis@veterinairespecialisten.nl; 2 Faculty of Science, School of Life and Environmental Sciences Camperdown, The University of Sydney, Sydney, NSW 2006, Australia; claire.wade@sydney.edu.au; 3 Expertise Center Veterinary Genetics, Faculty of Veterinary Medicine, Utrecht University, Yalelaan 104-106, 3584 CM Utrecht, The Netherlands; c.rozendom@uu.nl (C.R.); f.g.vansteenbeek@uu.nl (F.G.v.S.)

**Keywords:** dogs, cardiology, genetics, mitral valve disease, heart, murmur, breeding

## Abstract

The Cavalier King Charles Spaniel (CKCS) exhibits an unusually high prevalence of myxomatous mitral valve disease (MMVD). A potential link to MMVD for risk allele variants near the heart-specific nebulette (NEBL) gene has been identified. Although these risk allele variants seemed fixed in the CKCS, wild-type (i.e., healthy) allele variants at NEBL1-3 have likewise been found in a larger cohort, in which it was associated with less severe heart enlargement. The frequency of the wild-type allele variants in the asymptomatic breeding population is unknown. Therefore, the aim of this study was to investigate the wild-type allele variants frequency through prospective genetic testing in a large sample of CKCS that were intended for breeding in both the Netherlands and Australia. Blood samples of 370 CKCS with an unknown genetic status were collected, of which 175 from the Netherlands, and 195 from Australia. Deoxyribonucleic acid (DNA) was extracted for the genotyping of NEBL allele variants. No dog was homozygous for the wild-type allele variants. Only one dog from the Netherlands was heterozygous, while nine dogs from Australia were heterozygous. The prevalence of heterozygous dogs in the Australian breeding population was low (4.6%), but significantly higher compared to the prevalence in the Dutch breeding population (0.57%). In conclusion, selective breeding for the wild-type allele variants on its own would significantly reduce the number of breeding individuals and would add to the existing genetic bottleneck. The selective breeding of CKCS for wild-type allele variants should not be undertaken on its own due to the low prevalence in this breed and the polygenic character of the disease.

## Simple Summary

The Cavalier King Charles Spaniel is a dog breed in which mitral valve disease is very common. A potential link to this disease for risk allele variants near the heart-specific nebulette gene has been identified, where wild-type (i.e., healthy) allele variants are associated with less severe heart disease. The frequency of these wild-type allele variants in the asymptomatic breeding population is, however, unknown. Therefore, the aim of this study was to investigate the frequency of wild-type allele variants through genetic testing in dogs that were intended for breeding in both the Netherlands and Australia. No dog was homozygous (carrying two identical copies) for the wild-type allele variants. Only one dog from the Netherlands was heterozygous (carrying one wild-type and one risk allele variant), while nine dogs from Australia were heterozygous. The prevalence of heterozygous dogs in the Australian breeding population was low (4.6%), but significantly higher compared to the prevalence in the Dutch breeding population (0.57%). In conclusion, selective breeding for the wild-type allele variants on its own would significantly reduce the number of breeding individuals and would add to the existing genetic bottleneck. The selective breeding of Cavalier King Charles Spaniels for wild-type allele variants should not be undertaken on its own due to the low prevalence in this breed and the polygenic character of the disease.

## 1. Introduction

Myxomatous mitral valve disease (MMVD) is the most common cardiac disease in domestic dogs. The disease is characterized by slow progressive degenerative valvular changes, resulting in regurgitation. Subsequently, left atrial and ventricular dilatation might develop [1]. In some affected dogs, long-standing left-sided volume overload will ultimately progress to congestive heart failure [2]. Although the process of the disease has been well described [3], the underlying causes and mechanisms involved in the development and progression remain incompletely understood.

The Cavalier King Charles Spaniel (CKCS) is reported to be one of the most heavily bottlenecked domestic dog breeds, presumably because of multiple decades of closed population breeding and a small founder population [4]. As a result, CKCSs carry up to 13% more derived alleles than other dog breeds [4]. This is believed to contribute to high-risk states for certain diseases, including MMVD [5]. Murmur probability and severity, which indicate disease progression, have been suggested to be heritable in the CKCS [5,6]. Pedigree analysis suggests polygenic inheritance [6].

Genetic variation in allele frequencies between several breeds has been demonstrated using whole-genome sequencing data [4]. In CKCSs, a higher frequency was found of ten allele variants, of which six variants were located within the heart-specific nebulette (NEBL) gene. It was demonstrated that several of the NEBL variants had regulatory potential in heart-derived cell lines and were associated with reduced NEBL isoform nebulette expression in papillary muscle. Additionally, this study found that the genotype for one of the NEBL risk variants (NEBL3) was a significant predictor of graded MMVD status in the Dachshund.

Although these allele variants seemed fixed in the CKCS, wild-type (i.e., healthy) alleles at NEBL have likewise been demonstrated. A separate research group looked at risk variants in a large group of Australian CKCSs with different stages of MMVD [7]. The homozygous NEBL1-3 variants were associated with more severe cardiac dilatation, compared to the heterozygous genotypes. This finding suggests that dogs that carry the NEBL1-3 wild-type have less severe disease. In this study, the prevalence of the heterozygous genotype was low (3.4%).

Breeding restrictions have been shown to decrease the prevalence of MMVD [8,9]; therefore, breeding programs require or advise yearly auscultation or echocardiography for the screening of heart murmurs in CKCSs. In the Netherlands, for example, yearly auscultation is obligatory to obtain a registration number. Dogs that develop a murmur before 5 years of age are excluded from breeding in the Netherlands, with the intention of increasing the age of disease onset, similar to breeding programs in other countries [8,9].

The identification of breed-specific genetic mutations can alter breeding and screening recommendations for CKCSs. Developing more effectively targeted screening and risk stratification could be beneficial. Understanding the frequency of these NEBL variants allows for more informed breeding practices to potentially reduce the severity of MMVD while maintaining genetic variability. The frequency of the wild-type allele in the asymptomatic breeding population is, however, unknown. On one hand, MMVD severity in the registered CKCS breed population could be reduced via selective breeding with dogs that carry the wild-type allele. On the other hand, if the wild-type frequency in the breeding population is very low and the NEBL variant is proven to be causative for MMVD risk and/or severity, crossbreeding would likely be required to breed out severe MMVD in the CKCS.

Therefore, the aim of this study was to investigate the wild-type allele frequency through prospective genetic testing in a large sample of CKCSs that were intended for breeding.

## 2. Materials and Methods

### 2.1. Animals

Blood samples were prospectively obtained from apparently healthy CKCSs from Dutch and Australian breeding populations that were presented by their breeders for auscultation. Irrespective of the auscultation status, blood was collected in all dogs, and deoxyribonucleic acid (DNA) was extracted for the genotyping of the NEBL risk alleles. Sampling and auscultation were performed by a Diplomate of the European College of Veterinary Internal Medicine Companion Animals (ECVIM-CA) cardiology, with ethical approval from the Dutch “Instantie voor Dierenwelzijn Utrecht” (protocol number 16205-1-04) or the approval from the University of Sydney Animal Ethics Committee (approval numbers 2015/902 and 2018/1449). Each sample consisted of 1 to 2 mL of whole blood in ethylenediaminetetraacetic (EDTA) tubes and was stored at −18 °C. As part of this research project, an echocardiogram was not performed in the dogs.

### 2.2. DNA Extraction

For the Dutch samples, an automated extraction system (MagCore^®^, RBC Bioschience, New Taipei City, Taiwan) was used for extracting total DNA from whole blood samples. DNA quality was assessed with a NanoDrop^®^ spectrophotometer (Isogen Life Science, Utrecht, The Netherlands). For the Australian samples, 15–40 µL of thawed whole blood was incubated with 45 µL QuickExtract TM (Lucigen®, Middleton, WI, USA) at 65 °C for 15 min and then at 98 °C for 5 min, followed by dilution with water to a volume of 450 µL. The samples were centrifuged at high speed (approximately 15,000× *g*) for one minute to pellet the cell fragments and Polymerase Chain Reaction (PCR) was carried out using the supernatant.

### 2.3. Genetic Testing

For the Dutch samples, the primers for nebulette variants NEBL1, NEBL2, and NEBL3 were extrapolated from previous research [6] and are shown in Table 1.

**Figure 1:**
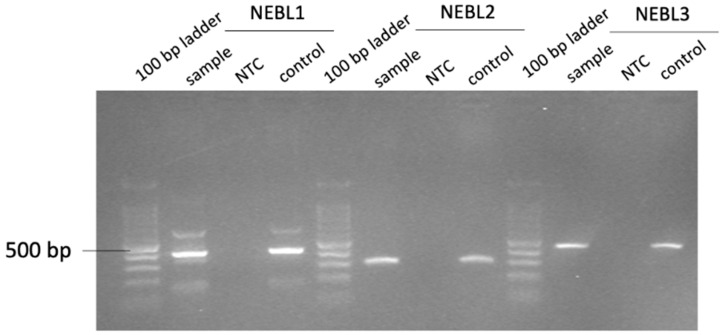
Gel electrophoresis with a 1.5% agarose gel and a 100 base pair ladder, under an ultraviolet light transilluminator (gel doc). Fragment NEBL1 is ±417 base pairs long, NEBL2 is ±277 base pairs long, and NEBL3 is ±429 base pairs long. NEBL, nebulette; NTC, negative control; control, positive control.

For each nebulette variant, a master mix, using Platinum Taq polymerase (Invitrogen by Thermo Fisher Scientific, Carlsbad, CA, USA), was prepared with the respective primers. A total of 25 ng/µL of the extracted DNA was added to the mix. A no-template control (NTC) was added as a negative control, and a known sample was used as a positive control. The PCR program was as follows: 5 min of denaturation at 95 °C, followed by 35 cycles of amplification for 30 s at 95 °C, 30 s at 57 °C (annealing temperature), and 30 s at 72 °C, followed by elongation for 10 min at 72 °C. The PCR product was checked for contamination using agarose gel electrophoresis (Figure 1). The PCR products were purified (to remove leftover primers) by adding 2U exonuclease I and incubated at 37 °C for 45 min followed by the deactivation of the enzyme by incubating at 75 °C for 15 min. Next, a BigDye sequencing reaction was performed using 1.5 µL BigDye Terminator 5X Sequencing buffer (Applied biosystems by Thermos Fisher Scientific, Waltham, MA, USA), 0.8 µL BigDye™ Terminator v3.1 Ready Reaction Mix, 4.7 µL of MilliQ, 2 µL of purified PCR-product, and 1 µL of 3.2 pmol of NEBL1 reverse primer, NEBL2 forward primer, or NEBL3 forward primer. The PCR program was as follows: 5 min of denaturation at 96 °C, followed by 35 cycles of amplification for 30 s at 95 °C, 15 s at 55 °C, and 30 s at 60 °C. This was followed by a purification step to remove unincorporated nucleotides using a gel filtration medium (Sephadex G-50, Cytvia, Wilmington, DE, USA). Sanger DNA sequencing was performed using the ABI 3500XL Genetic Analyzer (Thermo Fisher Scientific, Waltham, MA, USA). Data were analyzed using Lasergene Seqman Pro software (DNASTAR, Inc., Madison, WI, USA).

For the Australian samples, analysis was performed after the results of the Dutch samples were known. Since functionally significant variants of NEBL1-3 are in perfect LD based on earlier research [6], as confirmed by the results from the Dutch samples (all 175 Dutch samples had the same genotype at NEBL1, NEBL2 and NEBL3), only the NEBL3 variant was analyzed. PCR was performed with 7 µL supernatant combined with 7 µL primer mixture and 11 µL EconoTaq PLUS GREEN 2X master mix (Lucigen^®^, Middleton, WI, USA), and 32 cycles of competitive endpoint PCR were carried out with an annealing temperature of 52 °C. All animals were tested anonymously in location-randomized duplicates, and in all cases, there was agreement between the genotype indicated by the two duplicates. The primer mixture (Table 2) consisted of three primers, including a common reverse primer and two allele-specific forward primers. An extra “tail” was added to one of the allele-specific primers to create a larger fragment, which in turn facilitated discriminating between the two alleles either via gel electrophoresis or mass spectrometry. The Single-Nucleotide Polymorphism (SNP) being tested for was the final base on the 3′ end of the two forward primers. An additional mismatch to the genomic sequence was added within the 5 bases at the 3′ end of the primer. The deliberate inclusion of a mismatch in this manner greatly increases the specificity of each allele-specific primer and allows them to be tested in a competitive manner in a single PCR. The fragment lengths in this assay were 74 bp/94 bp.

### 2.4. Statistical Analysis

Data were analyzed with commercially available software (Microsoft^®^ Excel). Results are reported as median values with interquartile ranges (IQRs) or frequencies with a 95% confidence interval. Parameters between cohort types were analyzed with Fisher’s Exact test. Correlation between murmur and genotype was also analyzed with Fisher’s Exact test.

## 3. Results

### 3.1. Dutch Cohort

A total of 175 samples of CKCSs with an unknown genetic status owned by 38 different breeders (number of dogs per breeder: 1 to 17 dogs) were included (Table 3). The majority were female (36 male/139 female). The median age was 4 years with an interquartile age range between 2 and 6 years. A left apical systolic murmur indicative of MMVD was identified in 24 dogs (murmur prevalence: 13.7%). A total of 174 dogs were found to be homozygous for the NEBL1-3 risk allele variant. Only one individual was heterozygous, this was an 8-year-old female CKCS without a murmur. The heterozygosity prevalence in the Dutch breeding population was therefore 0.57% (95% confidence interval: <0.01% to 3.49%). No dog was homozygous for the wild-type allele variant. This resulted in a NEBL1-3 risk allele variant frequency of 99.7% (95% confidence interval: 98.2% to >99.9%).

### 3.2. Australian Cohort

A total of 196 samples of CKCS with an unknown genetic status owned by 66 different breeders (number of dogs per breeder 1 to 13 dogs) were analyzed (Table 3). One sample was excluded after DNA testing due to suspected contamination. Their age distribution was similar to the Dutch cohort, with a median of 4 years and an interquartile age range between 2 and 6 years. The majority were female (56 male/139 female). A left apical systolic murmur indicative of MMVD was identified in 57 dogs (murmur prevalence: 29.2%). Of these 195 dogs, 9 were heterozygous, 7 female dogs were aged 1 to 4 years, and 2 male dogs were aged 3 and 6 years. Within these nine heterozygous dogs, in two dogs, a left apical systolic murmur was identified (murmur prevalence: 22.2%). The heterozygosity prevalence in the Australian breeding population was therefore 4.6% (95% confidence interval: 2.33% to 8.66%). No dogs were homozygous for the wild-type allele variant. The NEBL3 risk allele variant frequency of the Australian cohort was 97.7% (95% confidence interval: 95.6% to 98.9%).

Murmur prevalence in the Australian breeding population was higher compared to the frequency in the Dutch population (*p* value: 0.0004). The wild-type allele variant frequency of the Australian breeding population was higher compared to the frequency in the Dutch population (*p* value: 0.0224). Likewise, the prevalence of heterozygous dogs in the Australian breeding population was higher compared to the prevalence in the Dutch breeding population (*p* value: 0.0216).

### 3.3. Combined Cohort

Considering the entire dataset of 370 dogs, 2 out of 10 heterozygous dogs presented with a left apical systolic murmur (murmur prevalence: 20.0%), whereas 79 out of 360 homozygous dogs presented with a murmur (murmur prevalence: 21.9%); this difference is not significant. In addition, of all 81 dogs with a murmur, 2 dogs were heterozygous (heterozygositiy prevalence: 2.47%), whereas of all 289 dogs without a murmur, 8 dogs were heterozygous (heterozygosity prevalence: 2.77%); this difference is not significant (Table 4).

## 4. Discussion

Although small differences exist between different breeding populations, this study shows a high NEBL risk allele variant frequency and homozygosity in CKCS breeding dogs in both the Netherlands and Australia. This is not surprising given the internationality of dog breeding and since dog breeds are created by a small number of founders combined with closed studbooks [10]. When a population size decreases for one or more generations, this is called a genetic “bottleneck”. From this small founder group, a new population is created that possesses reduced genetic variation with different allele frequencies compared to the ancestral population [11]. Deleterious recessive alleles will not show in heterozygous animals (the so-called masked load), but selection in small populations will increase homozygosity, which leads to higher expression of deleterious recessive alleles. The masked load becomes a “realized load” with disease expression [12]. Therefore, dogs generally carry more deleterious alleles in a homozygous state than wolves [13].

The formation of the CKCS breed has been linked to significant allele frequency shifts [4], with a percentage of inbreeding that has been estimated at about 40% [10,14]. The CKCS breed was revived in the United Kingdom in the 1920s with the aim of recreating the dogs shown in the paintings of King Charles II. Many CKCS kennel clubs and books refer to Roswell Eldridge who held a competition in 1926, looking for dogs as shown in the pictures of King Charles II [15]. Few dogs were selected to fit the standard. Details on the original gene pool and evidence on how the breed was recreated are lacking. In 1928, several breeders created the first CKCS kennel club in the United Kingdom. Descendants of these dogs spread throughout the world, forming the breeding populations in both the Netherlands and Australia, which likely have the same genetic characteristics.

Nebulette is a cytoskeletal structural protein found abundantly in cardiac myocytes [16], where it interacts with the thin filaments and Z-line associated proteins [17]. NEBL risk variants are associated with decreased nebulette expression in the papillary muscle, suggesting that the papillary muscle function has an important role in the development and/or progression of the disease [4]. The weakening of the papillary muscle could lead to mitral valve prolapse during systole, which is an important finding in MMVD.

The results of our study agree with previous findings stating that heterozygous genotypes are rare [4,7]. These dogs still develop MMVD, as also demonstrated in this study with similar murmur prevalence in homozygous versus heterozygous dogs. Moreover, murmur prevalence was more common in the Australian breeding population, while the prevalence of heterozygous dogs in the Australian cohort was higher than the prevalence in the Dutch breeding population. It seems, therefore, likely that MMVD is a complex trait [18], with genetic variants at the nebulette locus being associated with MMVD severity or progression [7], and that there are other contributing genetic factors [19], as previous research has also suggested that it is a polygenic disease [6]. In support of this, mitral valve transcriptome analysis in CKCS with or without heart failure identified 56 genes that were differentially expressed [20]. To better understand the genetic cause of this disease, further research is required. In this context, a recent study identified nine candidate genes that are associated with longevity in CKCS dogs, where the NEBL gene is not one of them [21]. A variant in Collagen Type XIX Alpha 1 Chain (COL19A1) was found, which encodes for a protein involved in cartilage strength and function. Collagen has been shown to play a crucial role in the development of MMVD. The health status of these dogs was not evaluated, so they did not compare dogs with MMVD with those without.

This study has several limitations. First, the NEBL risk allele variant frequency in breeding CKCSs from only the Netherlands and Australia was investigated. Although unlikely, given the common ancestry and international character of dog breeding, it is possible that in other countries or regions, the prevalence of the wild-type allele variants is higher. Second, analysis was performed using genetic variants at the NEBL locus. A causative mutation in the NEBL gene has not been identified yet, which should be a more tightly correlated marker. Third, there was a significant predominance of female dogs in our study, which was likely related to the larger number of female dogs used for breeding. It is possible that the prevalence of the wild-type allele variants might be different if sex distributions were more equal. Fourth, an echocardiogram was not performed in the dogs as part of this research project. It is therefore possible that in some of the dogs, left apical systolic murmur was not secondary to MMVD, whereas in some dogs without a murmur, mild MMVD might have been missed.

## 5. Conclusions

In conclusion, although small differences between countries exist, this study shows a high NEBL risk allele variant frequency and homozygosity in CKCS breeding dogs. Therefore, the selective breeding of CKCSs for wild-type NEBL allele variants should not be undertaken on its own due to the low prevalence in this breed and the polygenic character of the disease.

## Figures and Tables

**Table 1:** Sequences for primers used in PCR for the samples collected and analyzed in the Netherlands. PCR, Polymerase Chain Reaction; NEBL, nebulette.

Primer	Sequence 5′-3′
NEBL1_Forward	5′-GGAAGCAGGCTCAGACTCTC-3′
NEBL1_Reverse	5′-AACCTGACCAGTCCTTGGTG-3′
NEBL2_Forward	5′-GCAGAAGGGCAACACTCTCT-3′
NEBL2_Reverse	5′-TCTCTTTCTTTTGCCGCCCT-3′
NEBL3_Forward	5′-AGCCCTCCTTCTGTGCTTTA-3′
NEBL3_Reverse	5′-CTCCAAGGAGCCATCACATT-3′

**Table 2:** Sequences for primers used in the PCR for the samples collected and analyzed in Australia. PCR, Polymerase Chain Reaction.

Primer	Sequence 5′-3’
NEBL3_Forward (wild-type allele)	5′-CAGCAGACCCCAGCAGAGCG-3′
NEBL3_Forward (risk allele)	5′-AACTGATAGATCGGAATGGTCAGCAGACCCCAGCAGAATA-3′
NEBL3_Reverse	5′-TACAGAGTTTCAGTCCTGCCAAA-3′

**Table 3:** Numbers and proportions of the two cohorts of dogs used in the study. Different superscripts denote a significant difference between cohorts.

	Dutch Cohort (175 Dogs)	Australian Cohort (195 Dogs)
Age (Median; IQR)	4 years (2–6 years)	4 years (2–6 years)
Male/female (%)	20.0%/80.0%	28.7%/71.3%
Murmur prevalence (%)	13.7% ^1^	29.2% ^2^
Prevalence of heterozygous dogs	0.57% (1 female) ^1^	4.6% (7 females/2 males) ^2^

**Table 4:** Combination of all numbers and proportions of all dogs in this study.

	Homozygous for the Risk Allele	Heterozygous	Total
Left apical systolic Murmur (Yes)	79	2	81
Left apical systolic murmur (No)	281	8	289
Total	360	10	370

## Data Availability

Data are contained within the article.

## Abbreviations

The following abbreviations are used in this manuscript:

CKCS	Cavalier King Charles Spaniel;
COL19A1	Collagen Type XIX Alpha 1 Chain;
DNA	Deoxyribonucleic acid;
ECVIM-CA	European College of Veterinary Internal Medicine Companion Animals;
EDTA	Ethylenediaminetetraacetic acid;
IQR	Interquartile range;
MMVD	Myxomatous mitral valve disease;
NEBL	Nebulette;
NTC	No template control;
PCR	Polymerase Chain Reaction;
SNP	Single Nucleotide Polymorphism.
